# Restricting sports participation on public health and individual well-being grounds: a critical analysis of sport-related rights

**DOI:** 10.1007/s40318-025-00312-0

**Published:** 2025-09-09

**Authors:** Giulia Sesa, Pascal Borry, Sigmund Loland, Silvia Camporesi

**Affiliations:** 1 Department of Public Health and Primary Care, Centre for Biomedical Ethics and Law, KU Leuven, Leuven, Belgium; 2 Department of Sport and Social Sciences, Norwegian School of Sport Sciences, Oslo, Norway

**Keywords:** Sport participation restrictions, Eligibility, Rights, Public health, Individual well-being

## Abstract

Discussions on restricting participation in sports on public health and individual well-being grounds are not new to the sporting context. Over the past decade, such restrictions have ranged from containing infectious diseases to excluding athletes with noncommunicable diseases. This article addresses the question of whether and to what extent it is ethically and legally defensible for sport bodies and governments to impose such limitations through the lens of human rights, a crucial framework in public health ethics. In doing so, an analysis of normative instruments was performed to understand the articulation of sports-related rights in both sport-specific and non-sport-specific international rights declarations. The analysis yielded four primary observations: (1) an inconsistent depiction of sports as a right; (2) a broad and ambiguous definition of sports solely linked to positive values; (3) an absent differentiation between levels of participation; and (4) variations in the origin, scope, and binding nature of instruments. Such imprecision hinders the effective implementation and comprehension of the rights articulated in and the values upheld within these instruments and creates a fragmented legal landscape. Our work contends that the recognition of rights in relation to sports is fundamentally unrelated to debates concerning eligibility and public health in organized sports at all levels. The most comprehensive rights to participate in sports are stated at the most basic level of participation, specifically granting individuals the right to take part in recreational and leisure pursuits. Greater conceptual clarity is needed to resolve ambiguities and strengthen protections for individuals across all levels of sport.

## Introduction

Discussions on restricting sports participation based on public health and individual well-being concerns are not new to sports federations and the sporting context.^1^ Indeed, over the past decade, there have been numerous occasions where participation has been limited by governmental bodies and sports federations. These restrictions have been due to a variety of reasons, ranging from the containment of infectious diseases to excluding elite and professional athletes with heart conditions, relative energy deficiencies (REDs), and other chronic and infectious diseases from competing in sports, as well as temporarily suspending athletes following sport-related injuries (e.g., concussions).^2^

The COVID-19 pandemic stands out as a significant example. While active living was encouraged to mitigate lockdown-related health impacts, performing sports was perceived as risky behavior. The pandemic led to the halting of sports events and competitions, the shutdown of fitness establishments, and the imposition of constraints on outdoor sports.^3^ Across many European and non-European countries, the opportunity to participate in sports was largely restricted to professional athletes with sportsmen and women being allowed to train, compete, and travel across borders within tightly controlled ‘*sporting bubbles*’.^4^ For instance, professional football players were permitted to resume training and participate in matches behind closed doors, while more stringent restrictions were applied to amateur sporting activities.

Discussion on HIV/AIDS also led to the introduction of limitations to sports participation. Athletes who tested positive for HIV often encountered professional obstacles, including exclusion from competitions.^5^ This has been the case for well-known sports personalities Earvin ‘Magic’ Johnson, professional basketball player, and Tommy Morrison, professional boxer and mixed martial artist. Recent years have seen a growing discourse on athletes with heart conditions barred from sports.^6^ For instance, Christian Eriksen’s cardiac arrest and subsequent defibrillator implantation led to his disqualification from Serie A due to the league’s health regulations, forcing him to leave Italy and relocate to the UK to sustain his professional football career. Most recently, the International Federation of Sport Climbing (IFSC) established a new benchmark by being the first international federation to adopt comprehensive regulations concerning relative energy deficiencies in sport (REDs) in February 2024.^7^ Sports federations have also implemented protocols to address injuries like concussions, which may include temporary restrictions on participation, or, in cases of recurring injuries, extended suspensions based on medical assessments—sometimes lasting an entire season. In combat sports such as boxing and mixed martial arts, a knockout or technical knockout typically triggers an automatic medical suspension.^8^ Similarly, under Rugby Australia’s Concussion Management Procedure, players who sustain more than three concussions in a single season are strongly advised not to return for the remainder of that season.^9^

Sports participation and eligibility decisions vary greatly across different sports federations, leading to a lack of clarity. This is further complicated by the ethically sensitive nature of public health policy. Here, professionals and policymakers are constantly balancing collective and individual well-being, rights, and needs.^10^ These complexities highlight the need for further research.

This article aims to address the question of whether and to what extent it is ethically and legally defensible for sport bodies and governments to limit sports participation on public health and individual well-being grounds through the lens of human rights, a crucial framework in public health ethics.^11^ Firstly, it presents an analysis of normative instruments that recognize rights in relation to sports. Specifically, it undertakes a critical analysis of the quality, precision, and relevance of such instruments in the context of restricting sports participation on public health and individual well-being grounds. Next, it discusses how exclusion from participation in sports aligns with the principles and viewpoints defined in international instruments expressing rights in relation to sports, outlining the implications of different formulations. Particularly, this article also explores the right to take part across various levels of participation, including recreational, grassroots, elite, and professional sports. By adopting a broader ethical and legal perspective–grounded in the analysis of international human right instruments–the findings of this research will be particularly relevant to both legal and ethical scholars, as well as policymakers and practitioners in the legal and public health fields, who navigate the balance between public health measures and individual rights in relation to sports.

## Materials and methods

A document analysis was performed to investigate the articulation and framing of sports-related rights in both sport-specific and non-sport-specific international rights instruments stemming from the Universal Declaration of Human Rights and sports-related charters such as the Olympic Charter. The READ approach—(1) ready your materials, (2) extract data, (3) analyze data, and (4) distil your findings—suggested by Dalglish et al. (2020), was utilized as a systematic technique for scrutinizing documents and extracting relevant data.^12^

To identify relevant sport-specific and non-sport-specific international rights instruments—step 1 of the READ approach—a literature search was first conducted across multiple databases (e.g., Google Scholar, PubMed, ScienceDirect, and Taylor & Francis Online) using keywords such as ‘rights,’ ‘sport,’ ‘physical activity,’ ‘leisure,’ ‘participation,’ and ‘access’ during March–April 2024. Moreover, the citations and reference lists of the retrieved articles were examined to identify additional relevant articles on rights associated with sports, with particular attention to references that were consistently cited across multiple sources. This approach served as a method to sample relevant rights-related instruments and to ensure that no pertinent ones were omitted from the analysis. The sampling of articles ceased once it became evident that scholarly articles consistently echoed the same international rights instruments without introducing any new ones.

Instruments were included in the analysis if they were internationally scoped and included sport-related rights. The definition of ‘sport’ and ‘sports-related rights’ was broad to ensure comprehensive identification of relevant instruments. ‘Sport’ was defined as any physical activity involving both casual and organized participation, encompassing but not limited to competitive activities governed by rules. ‘Sports-related rights’ were understood as encompassing various formulations including participation, access, and the acknowledgment of sports practice as a right. Additionally, ‘sports-related rights’ were also interpreted as covering those rights that, while not directly linked to sports, provide a framework under which sports-related rights can be protected and promoted, viz. cultural rights. Legal instruments associated with national rights were excluded from the analysis. United Nation’s (UN) instruments were included in the analysis given their global relevance. European instruments like the European Charter of Fundamental Rights and the European Convention on Human Rights were also included given that most sports federations are located in Europe and thus subject to EU Law.^13^ Additionally, sport-specific instruments, such as the Olympic Charter, were incorporated into the analysis due to their foundational role in shaping the governance and principles of the sports domain. Table 1 provides an overview of the types of documents included in the study, outlining their purpose, the inclusion and exclusion criteria applied, and the final number of instruments included for analysis.

The extraction and analysis of data emerging from the retrieved normative instruments– steps 2 & 3 of the READ approach—followed the stages described in the article by Bowen (2009), namely skimming, reading, and interpretation.^14^ Passages of the texts were deemed relevant if they recognized sports as a right, outlined duties of public and/or sports authorities in relation to sports, provided relevant definitions of sports, or highlighted rights for specific vulnerable groups. Such passages were highlighted with different colors and coded according to six categories with the keywords: rights, duties, definition, benefits, and vulnerable populations. A careful and more focused re-reading of the coded documents then followed to critically reflect on the framing of sports as a right, on the applicability of the instruments to different levels of sports, on the definition of sports, and on similarities and differences across instruments—step 4 of the READ approach.

**Table 1:** Type of documents included in the analysis and inclusion and exclusion criteria

Source type	Purpose in the study	Inclusion criteria	Exclusion criteria	Outcome
Academic Literature	To identify rights-related instruments and provide conceptual/theoretical background to support arguments	Peer-reviewed articles English Language Discussing human rights and sports Published up to April 2024	Non peer-reviewed sources (i.e., gray literature and media articles). Articles without reference to rights instruments	The identification of relevant instruments followed a two-step process. First, a literature search was conducted to identify scholarly articles discussing sport-related rights. Second, reference lists and frequently cited works were examined to locate additional relevant articles. The identified scholarly articles were reviewed to extract sport-specific and non-sport-specific rights instruments cited within them.
Sport-specific and non-sport specific international rights instruments	Main objects of analysis—READ approach	International scope (viz. UN and EU instruments) Explicit or implicit reference to sport-related rights Sport-specific instruments at the international level	National legal instruments Local sport-specific instruments	Fifteen sport-specific and non-sport-specific rights instruments were included in the analysis, identified through the literature search and selected according to the established inclusion and exclusion criteria.

## Normative instruments implicitly affirming rights in relation to sport

As suggested by Veal (2022),^15^ the idea of sports as a human right is supported by Article 24 of the Universal Declaration of Human Rights (UDHR), which guarantees everyone “*the right to rest and leisure*,” and Article 27(1), which ensures “*the right freely to participate in the cultural life of the community*”.^16^ Such rights are reaffirmed in Articles 7 and 15 of the International Covenant on Economic, Social and Cultural Rights (ICESCR), which respectively recognize “*the right of everyone to the enjoyment of just and favorable conditions of work*” including *“(d) rest, leisure and reasonable limitation of working hours…*” and “*the right of everyone: (a) to take part in cultural life*”.^17^

Reflecting on the definitions of culture and leisure is crucial for ascertaining whether such articles and associated rights can be interpreted to include participation in sports. According to the United Nation’s Committee on Economic, Social, and Cultural Rights (2009), culture is an expansive and inclusive concept that covers all forms of human expression and existence.^18^ Precisely, culture refers to “*ways of life, language, oral and written literature, music and song, non-verbal communication, religion or belief systems, rites and ceremonies, sport and games, methods of production or technology, natural and man-made environments, food, clothing and shelter and the arts, customs and traditions through which individuals, groups of individuals and communities express their humanity and the meaning they give to their existence”*.^19^ Conversely, the concept of leisure is not formally defined in the UDHR or ICESCR. These instead rely on an intuitive understanding of leisure, associating it with time not dedicated to paid work—although it could be described more precisely as non-obliged time—and involvement in cultural activities.^20^ The World Leisure Organization (WLO) Charter for Leisure is a crucial resource to reference and examine when considering the rights of individuals to leisure and investigating how this right relates to sports. The most recent 2020 Edition of the Charter declares that *every person, irrespective of age, has the right to adequate rest and leisure pursuits* (Article 1), *as well as the liberty to partake in the cultural activities of their community* (Article 4)*. This includes involvement in music, ceremonies, sports, and interactions with both the natural and man-made surroundings*. Therefore, sports, outdoor activities, and play are considered part of leisure.^21^

In addition to the UDHR, the ICESCR, and the WLO’s Charter of Leisure, a variety of international instruments, both linked to and separate from the United Nations, affirm the right of individuals or particular population groups to engage in cultural and leisure activities. These include, for instance, the 1996 European Social Charter (Articles 15, 23, and 30), the 1965 International Convention on the Elimination of All Forms of Racial Discrimination (Article 5(e) (vi)), the 1989 United Nations Convention on the Rights of the Child (UNCRC) (Article 31(1)), the 1979 Convention on the Elimination of All Forms of Discrimination against Women (CEDAW) (Article 13(c)), the 2006 Convention on the Rights of Persons with Disabilities (CRPD) (Article 30); the 1992 Declaration on the Rights of Persons Belonging to National or Ethnic, Religious and Linguistic Minorities (Article 2(1)(2)), and the 1990 International Convention on the Protection of the Rights of All Migrant Workers and Members of Their Families (Article 43(1)(g)). By acknowledging the right to culture and leisure, these instruments indirectly endorse sports as a human right by the definitions of culture and leisure illustrated in the previous paragraph. Additionally, some of these instruments explicitly endorse sports as a distinct human right (*see *section 4—*legislative instruments explicitly affirming sports as a human right*).

Delaney and Madigan (2023) argue that sports are essential for achieving what they refer to as ‘*eudaimonia’*, or a fulfilling life. By considering sports as indispensable for a well-rounded life and the enhancement of one’s intellectual, physical, and cultural development, and considering the interdependence of human rights, it can be argued that sports (at the most basic level of participation) are implicitly recognized as a right by Articles 3 and 25(1) of the UDHR, Articles 2(1) and 35 of the Charter of Fundamental Rights of the European Union (2012) (CFR), and Article 2 of the European Convention on Human Rights (1950). These articles guarantee respectively one’s right to life and one’s right to an adequate standard of living for health and well-being—as formulated in the UDHR. Additionally, Article 3(1) of the CFR upholds that “*Everyone has the right to respect for his or her physical and mental integrity*”. While rights in relation to sports are not explicitly mentioned in the Charter, the practice of sports can be seen as a way to respect and uphold one’s physical and mental integrity, which is protected by this article. Moreover, in the context of professional athletes, sports may be considered a human right by being part of their broader right to work, outlined in Article 23(1) of the UDHR, Article 15 of the CFR, and Article 6(1) of the ICESCR. This latter states that: “*States Parties to the present Covenant recognize the right to work, which includes the right of everyone to the opportunity to gain his living by work which he freely chooses or accepts, and will take appropriate steps to safeguard this right*”. Ultimately, it has been suggested that there could be a correlation between sports access rights and educational rights, as acknowledged under international law.^22^ Specifically, schools are key providers of sports opportunities and play a crucial role in ensuring access to sports, helping children’s intellectual, physical, and moral development.^23^ Access to sports could therefore be upheld through the broader right to education (*see *section 4—*legislative instruments explicitly affirming sports as a human right*).

Finally, it is crucial to recognize that the UDHR does not legally bind states, nor does it enforce sanctions or commitments under international law. In contrast, the ICESCR compels its signatories to actively work towards the rights it enumerates, using “*the maximum of its available resources*” for their full realization (Article 2).^24^ Likewise, the CFR and the European Convention on Human Rights are legally binding.

The ICESCR outlines three principal obligations for state parties concerning the rights to leisure and culture: the obligations to respect, protect, and fulfil. The former obligation, “*requires States parties to refrain from interfering, directly or indirectly, with the enjoyment of the right*”.^25^ The obligation to protect “*requires States parties to take steps to prevent third parties from interfering in the right to take part in cultural life*”.^26^ Ultimately, the obligation to fulfil, “*requires States parties to take appropriate legislative, administrative, judicial, budgetary, promotional and other measures aimed at the full realization of the right*”.^27^ In line with these obligations, the WLO Charter for Leisure recognizes the duties of governments for upholding the right to leisure and culture. Specifically, it acknowledges in Article 7 the duties of local, regional, and national governments to, among other things, provide services and facilities for leisure, guarantee the availability of appropriate spaces and facilities for children’s play, endorse the creation of amenities that promote health, like sports and exercise facilities, and provide adequate training for a skilled workforce in the leisure, sports, and cultural service sectors.^28^ Additionally, it emphasizes the importance of non-discriminatory access to leisure facilities and services, the necessity of upholding human rights that enable involvement in the community’s cultural activities, as well as the incorporation of leisure-related rights within the applicable national or provincial legal frameworks.^29^ Similarly, the UN Committee on Economic, Social and Cultural Rights identifies the presence of accessible, affordable, acceptable, and appropriate “*cultural goods and services that are open for everyone to enjoy and benefit from, including libraries, museums, […], sport stadiums*” as essential for the actualization of the right to participate in cultural life.^30^ Moreover, the Committee acknowledges that, although the primary duty to adhere to the Covenant lies with the States Parties, every segment of society bears a role in the actualization of the right for all to engage in cultural life.^31^ It is incumbent upon States parties to oversee and ensure the corporate sector and other non-state entities uphold this right.^32^

## Normative instruments explicitly affirming rights in relation to sport

The 1970s marked a significant turning point in the explicit recognition of rights in relation to access and participation in sports.^33^ In 1966, the Council of Europe introduced the concept of “*Sport for All*,” emphasizing that the practice of sports is a fundamental right for all.^34^ In 1976, the first European Sport for All Charter was adopted by the Committee of Ministers (1976) during the 26th meeting of the Ministers’ Deputies. This Charter, through eight articles, outlined a comprehensive framework for promoting “*Sport for All*” across Europe, being instrumental in the development of European sports policies and in enabling individuals to exercise their right to participate in sports.^35^ Notably, Article 1 affirms that “*every individual shall have the right to participate in sport*”. The 2001 revision of the Charter (Recommendation No. R(92)13 rev) affirms in articles 1, 4(2), and 6(1) the responsibilities of governments in enabling “*every individual to participate in sport*”, ensuring “*that all citizens have opportunities to take part in sport”*, and in promoting “*the practice of sport, […] for all parts of the population*”. Notably, the 2001 revision of the Charter was modified to incorporate a definition of sports, defining it as being “*all forms of physical activity which, through casual or organized participation, aim at expressing or improving physical fitness and mental well-being, forming social relationships or obtaining results in competition at all levels*”. This definition was also retained in the 2021 revision of the Charter. The 2021 Revised European Sports Charter explicitly recognizes the right to access (*rather than participate in*) sports, a right that was originally affirmed in the 1976 Charter but was not acknowledged in the 2001 revision. Article 10(1) states: “*Access to sport for all is considered to be a fundamental right. All human beings have an inalienable right of access to sport in a safe environment, […], which is essential for their personal development and instrumental in the exercise of the rights to health, education, culture and participation in the life of the community*”. Articles 1 and 10(3) mandate that governments must undertake the necessary actions to enable every individual to participate in sports. These measures include: ensuring access to sports and physical education for intellectual, physical, and moral development; providing inclusive and safe opportunities for all to engage in sports activities and physical education; fostering physical health and literacy, teaching essential movement skills, and helping individuals reach their potential; facilitating affordable participation in sports via suitable legal frameworks and status for local clubs; and enabling all individuals to elevate their performance levels beyond leisure purposes. Furthermore, Article 12(1) emphasizes the need to foster the practice of sports among the entire population by offering suitable facilities and programs, and ensuring access to skilled coaches, instructors, and staff. Particularly, according to Article 15(1), “*since participation in sport is dependent in part on the extent, the variety and the accessibility of facilities, their overall planning should be a matter for the public authorities*”.

Almost concurrently with the first European Sport for All Charter, the UNESCO International Charter of Physical Education and Sport was adopted at the 20th Session of the UNESCO General Conference in 1978. This Charter served as a pioneering tool for advocating global access to sports and aiding in the formulation of sports-related decisions and policies. Article 1 of the Charter explicitly declares that “*the practice of physical education and sport is a fundamental right for all*” and that “*every human being has a fundamental right of access to physical education and sport, which are essential for the full development of his personality”.* The article further stresses in its second and third paragraphs that all individuals should have ‘*full opportunities*’ to practice physical education and sports and that particular provisions must be established for vulnerable groups. Additionally, Article 5 emphasizes the importance of adequate equipment and facilities provision, noting that public authorities, governments, private agencies, and schools have to collaborate in the planning and ensuring the provision and optimal use of equipment and facilities for sports and physical education.

Likewise, the Revised International Charter of Physical Education, Physical Activity and Sport, adopted during UNESCO’s 38th session of the General Conference (2015), affirms in Article 1 that “*the practice of physical education, physical activity and sport is a fundamental right for all*” and that “*every human being has a fundamental right to physical education, physical activity and sport without discrimination […]*” (Article 1 (1)). It is noteworthy that the term physical activity has been incorporated into such an article, marking a shift from the wording of the same article in the Charter’s 1978 edition. This suggests a possible distinction between physical activity and sport. However, the Charter does not provide definitions for these terms. Article 1(2) further emphasizes the need for support on behalf of governmental, educational, and sports institutions in developing physical, psychological, and social well-being and capabilities through sports and physical activity. Specifically, “*inclusive, adapted and safe opportunities to participate in physical education, physical activity and sport must be available to all human beings*” (Article 1 (3)). Article 1(7) emphasizes the responsibilities of schools in providing “*quality and inclusive physical education classes […], preferentially on a daily basis, as a mandatory part of primary and secondary education*”, reinforcing that physical activity and sport must be integral to the daily routine of children and youth. Additionally, Article 3 outlines the roles of all stakeholders, at both the local and national levels, in supporting and developing policies related to physical education, physical activity, and sport. Lastly, Article 8(2) stresses the importance of collaboration between sports and public authorities as well as schools and institutions in designing, making available, and optimizing equipment and facilities for such activities.

In 1996, the Olympic Charter formally acknowledged sports as a human right. Such a right was officially incorporated as the 8th Fundamental Principle of Olympism, which affirms that: “*The practice of sport is a human right. Every individual must have the possibility of practicing sport in accordance with his or her needs*”.^36^ Although the core principles of Olympism have undergone revisions throughout the years, the acknowledgment of sports practice as a right has consistently been maintained in official documents ever since. Presently, it is established as the 4th Fundamental Principle of Olympism which states that: “*the practice of sport is a human right. Every individual must have access to the practice of sport, without discrimination of any kind in respect of internationally recognised human rights within the remit of the Olympic Movement*”.^37^ While the Olympic Charter’s formal recognition of a right to sports may seem belated relative to earlier instruments, it is crucial to emphasize that Pierre de Coubertin’s idea that sports “*is not merely a pastime for the affluent or a physical outlet for intellectuals*” predates this formal recognition dating back to the 1890s.^38^

Ultimately, the United Nations Convention on the Elimination of All Forms of Discrimination against Women (CEDAW), the Convention on the Rights of the Child (UNCRC), and the Convention on the Rights of Persons with Disabilities (CRPD) explicitly recognize a right to participate in sports for certain marginalized groups who have traditionally faced challenges in gaining access to sports activities.^39^ The CEDAW, adopted by the United Nations General Assembly in 1979, mandates in Articles 10 and 13 that State Parties abolish discrimination, allowing women “*the same opportunities [as men] to participate actively in sports and physical education*”, and affirming equal rights for both genders “*to participate in recreational activities, sports and all aspects of cultural life*”. The International UNCRC, adopted by the United Nations General Assembly—resolution 44/25—in 1989, acknowledges in Article 31(1), the “*right of the child to rest and leisure, to engage in play and recreational activities appropriate to the age of the child and to participate freely in cultural life and the arts*”. Lastly, the CRPD, adopted by the General Assembly by resolution A/RES/61/106 in 2006, mandates in article 30(5) equal participation opportunities for persons with disabilities in sports and leisure, requiring states to promote their inclusion in mainstream sports, provide opportunities for disability-specific activities, ensure access to venues and services for these activities, and guarantee equal access for children with disabilities in school and play.

## Clarifying the landscape: the compatibility of sports participation restrictions with normative rights frameworks

The question of whether rights in relation to sports participation exist is addressed differently by various authors, with some supporting the existence of such a right and others questioning it.^40^ This section discusses the potential conflict between restricting sports participation on public health and individual well-being grounds and the existing framework of rights pertaining to sports. Specifically, we argue that discrepancies in the portrayal of sports as a right across normative instruments, their different origin, scale, and binding nature, the absence of a definition of sports, the common framing of sports only in relation to its beneficial effects, the various tiers of sports participation—recreational, grassroots, elite, and professional—and how a right to sport would be interpreted at each level, and the autonomy afforded to the sports sector challenge the instrumentality of such instruments in eligibility debates.

To begin with, the above review of normative instruments uncovers inconsistencies in the portrayal of sports as a right. On the one hand, some instruments envision sports under the umbrella of social, economic, and cultural rights, while on the other hand, other instruments refer directly to the right to *practice, access, or participate in* sports. Although interconnected, the right to access sports, the right to participate in sports activities, and the acknowledgement of sports practice as a right are distinct notions and rights.

Acknowledging the right to access sports involves contextualizing this right with respect to the availability or physical access of fundamental sports facilities, equipment, and programs, as well as ensuring non-discriminatory access to these resources.^41^ Therefore, in defining the right to access sports, it is essential to consider factors such as the availability, accessibility, and affordability of sports facilities, equipment, and programs. The limited resources of states cannot be used to justify denying the right of access to sport, as Article 2(1) of the ICESCR requires each state party to fulfil this right to the maximum extent of its available resources.^42^ Contrastingly, defining participation in sports as a right goes beyond mere access to sporting facilities. It is meant to make sure that individuals can actively take part in sporting activities without being excluded or discriminated against. Moreover, participation has a broader scope, extending beyond athletes to include spectators and others involved in sports at all levels, while also encompassing inclusion in decision-making processes.^43^ The right to participate in sports thus supports the involvement of athletes and diverse voices in shaping sport eligibility and membership criteria, which is also a fundamental element in making ethically sound eligibility policies in sports.^44^ Lastly, the acknowledgement of the practice of sports as a right appears more challenging to articulate. This complexity arises because such right appears to be closely associated with sports access, suggesting that these terms are frequently used interchangeably with little differentiation.^45^ We would be prone to interpret this as an overarching right which entails providing individuals with the freedom to partake in recreational activities, thereby allowing them to engage in basic sporting activities such as running, walking, or cycling.

Based on the above definitions, we believe that the right to participate in sports emerges as the most relevant formulation for challenging exclusion in sports on public health and individual well-being grounds. This right is affirmed in the UNCRC, CRPD, and CEDAW, making these documents, in our view, the most instrumental references for eligibility debates. However, the relevance of such instruments remains questionable. Specifically, in expressing a right to participate in sports these do not do so directly but rather use equal opportunity terminology for groups that have traditionally been marginalized and excluded. On the one hand, “*the right to participate in sport and recreation has been regarded as a second-class right”* and its recognition in the CRPD signifies a crucial advancement in articulating social rights.^46^ In simpler terms, the inclusion of Article 30(5) in the CRPD impacted the rights not only of those with disabilities but of all people.^47^ Similarly, one could make the case for the CEDAW and the UNCRC, emphasizing how the equal opportunities to participate in sports for women and the rights of children to rest, leisure, and play may have broader implications across the population. Nonetheless, although various normative instruments protect individuals who face exclusion from sporting opportunities due to prohibited attributes–such as disability–, these do not necessarily translate into unequivocal support for a universal right to participate in sports.^48^ In certain contexts, particularly in competitive and elite sports, restrictions may still apply on discretion of sporting bodies and rarely subject to judicial intervention.^49^ In practice, such discretion may extend to cases where an impairment could confer a competitive advantage (as in the debates surrounding Oscar Pistorius and Markus Rehm) or where chronic health conditions—such as HIV or severe mental illness—pose safety concerns.^50^

Reflecting on the way that sports is conceptualized throughout these normative instruments is also crucial for assessing their relevance and instrumentality in eligibility issues on public health and individual well-being grounds. The reviewed instruments operate with a vague and extensive idea of sports. Typically, sports are defined as the process of training for and engaging in competitive activities governed by rules, where bodily movement is an integral and meaning-producing component.^51^ Although a formal definition of sports is not consistently provided in the majority of instruments reviewed, it appears that sports is framed as encompassing activities that extend beyond competition and organized participation. These include, for instance, casual engagement aimed at enhancing physical fitness, mental well-being, and social connections.^52^ Moreover, it is evident from the normative texts and common discourse that sports are frequently portrayed as beneficial for both individuals and communities. For example, Article 2 of the International Charter of Physical Education, Physical Activity and Sport states that *“physical education, physical activity and sport can yield a wide range of benefits to individuals, communities and society at large”*. Similarly, the preamble of the 2021 Revised European Sports Charter acknowledges that *“sport can make diverse contributions to personal well-being and social development, and physical exercise in particular helps to promote both physical and mental well-being”.*

Public policy often views sports as a means for health promotion. However, elite and professional sports can often be at odds with the ideals of pleasure, leisure, and well-being, leading for instance to acute and chronic injuries, and even hazing and abuse incidents.^53^ The focus in elite and professional sports is not on health promotion, but on performance and risk prevention, this latter also based on performance optimization (such as reducing the risk of injury through personalized training strategies). This discrepancy raises questions about whether these normative texts accurately reflect the nature of sports and raise concerns about their applicability to different levels of sports. Distinguishing among recreational activities, grassroots sports, and organized competitive sports is crucial due to differences in organization, objectives, and value profiles of the activities. The complexity of what falls under the umbrella of “sports” spans a wide spectrum—informal and formal, organized and unstructured, social and elite, individual, pair, and team-based, mental and physical, outdoor and indoor, static and highly mobile, rapid and endurance-based—making definitional clarity essential.^54^ This diversity extends to emerging activities such as esports, whose inclusion within the scope of “*sport*s” in normative instruments remains uncertain and warrants further research.^55^ This definitional uncertainty has practical consequences. For example, during COVID-19 restrictions, governments often permitted certain low-contact sports such as golf while prohibiting higher-contact team sports like football. From a human rights perspective, it remains unclear whether such targeted restrictions infringe upon a recognised “*right to sport*,” or whether the activities in question are even encompassed within the definition of sport under normative instruments—particularly given that individuals still had access to alternative forms of physical activity such as walking, cycling, or exercising at home in some contexts. Similarly, temporary exclusions due to medical conditions—such as concussions, relative energy deficiency, or cardiac risk—raise questions about whether these situations fall within the rights articulated in international instruments. However, the lack of clarity in the instruments examined hinders effective implementation and understanding of the rights expressed in, and the values upheld by, these instruments, making such cases challenging to resolve within the existing normative framework.

A basic protection to the most basic level of participation in sports can be inferred from the analyzed instruments. Indeed, the conceptualization of sports in normative texts, the rights expressed within them, and the obligations that derive from these rights, all indicate that the primary aim of such instruments is to foster individual growth through sports and encourage states to create an environment conducive to sports practice. From this, it follows that the acknowledgement of recreational/leisure activities as a right, or a right to physical movement or activity, deemed a part of cultural rights, applies to all. Therefore, when restrictions are imposed at the most basic level of sports participation, such as preventing a citizen from running or cycling a clear tension arises. However, public health emergencies represent an exceptional circumstance under which governments can limit certain rights. Any restriction on non-derogable human rights is permissible in accordance with Article 4 of the ICESCR if it serves a legitimate purpose, is based on legal grounds, and is essential for safeguarding society’s general welfare^56^. Furthermore, the International Health Regulations (IHR) and national derivatives—such as laws that allow for declaring a situation of national emergency—form a legally binding instrument that enables states to take actions that may compromise the rights of the individuals to protect the health of the public and the functioning of states in public health emergencies.^57^ Such limitations must, also according to public health ethics principles, be proportionate, and the least restrictive measure should always be prioritized.^58^

Although the analysis of normative instruments supports individuals’ right to partake in personal recreational physical activity pursuits, these do not safeguard against being barred from participation, training, or competition in organized sports at all tiers, such as becoming a member of an athletics or cycling team or taking part in the Olympic Games. Particularly, in grassroots sports, individuals have the right to participate in an activity of their choice. However, sports clubs retain the authority to determine the membership of athletes. Likewise, in elite and professional sports, the right to compete in events depend upon athletes meeting several requirements and conditions outlined in the Charter’s Eligibility Code, along with the sport’s specific eligibility criteria established by National Governing Bodies (NGBs), National Olympic Committees (NOCs), and International Federations (IFs).^59^ Those who fail to meet necessary qualification standards cannot logically demand participation at the professional or elite level.

The reality is that there is no absolute right to participate; therefore, athletes may participate in official sporting competitions only if they satisfy eligibility requirements specified in the rules and regulations devoted to certain sporting competitions.^60^

The diversity in the scope and binding nature of the cited instruments contributes to the complexity of enacting sports-related rights. Normative instruments such as the WLO Charter of Leisure, the European Sport for All Charter (in all versions), the UNESCO International Charter of Physical Education and Sport (1978), and the UNESCO International Charter of Physical Education, Physical Activity and Sport (2015) are non-binding and not legally enforceable. While they suggest actions for state parties, they are primarily intended as guidelines for best practices rather than legally binding obligations. Further complicating the issue is the differing origin of these instruments, ranging from the UN to the EU, as well as the unique case of the Olympic Charter, all of which influence their scope of applicability. UN instruments have a global reach, while EU instruments are primarily applicable within European jurisdictions. As the foundational instrument for the Olympic Movement, the Olympic Charter imposes binding obligations on sport bodies, including National Olympic Committees, International Sports Federations and the Organizing Committees for the Olympic Games through a series of interrelated contracts.^61^ This diversity in origins, scope, and binding nature creates a fragmented legal landscape, making the realization of sports-related rights particularly challenging.

A more coherent legal framework—whether through clarification of existing rights or the establishment of separate rights—would reduce fragmentation and strengthen protections for individuals at all levels of sports participation. Such clarification is also essential to enable states, as primary duty bearers, to assume a more central role rather than leaving eligibility decisions solely in the hands of sports bodies and shielded from external oversight due to the "*autonomy of sports*." As also emphasized by the Special Rapporteur Alexandra Xanthaki, neutrality in human rights matters is not applicable.^62^ However, the current lack of clarity makes this a difficult task.

## Conclusions

Our analysis of international instruments expressing rights in relation to sports yielded four primary observations: (1) an inconsistent depiction of sports as a right; (2) a broad and ambiguous definition of sports solely linked to positive values; (3) an absent differentiation between levels of participation; and (4) variations in the origin, scope, and binding nature of the analysed instruments. Such imprecision hinders the effective implementation and comprehension of the rights articulated in and the values upheld within these instruments and creates a fragmented legal landscape.

We contend that the recognition of rights in relation to sports in international rights instruments is fundamentally unrelated to debates concerning eligibility and public health in organized sports at the grassroots, elite, and professional levels. In practice, the essence of the referenced normative instruments appears to be the promotion of physical, intellectual, and social growth through sports, which is more in line with the idea that people have a right to recreational/leisure activities, bodily movement, or physical activity rather than sports. On this basis, states are required to create conditions that allow people to participate in sports, such as providing adequate facilities for everyone to benefit from, providing a skilled workforce involved in sports, and ensuring that there is no discrimination in access to sporting facilities. Thus, a distinct tension emerges when participation in sports is restricted at its most basic level for reasons of public and personal health. In organized sports, sporting organizations can, in the current context, determine the eligibility of prospective members with minimal regulatory oversight as long as they avoid discriminating based on prohibited attributes. To strengthen the protection for individuals at all levels of sports participation, further conceptual clarity is necessary*—*for instance by clarifying existing rights or by establishing separate rights specific to sport.

This work presents several limitations to be addressed in future research. First, the question of whether it is ethically justifiable to restrict participation in sport extends beyond a human rights framework. Rights may be limited in certain circumstances, such as for public health protection during a pandemic, requiring consideration of additional ethical frameworks, including public health ethics frameworks that balance collective and individual (health) interests. Second, while this analysis adopts an international focus, it reflects a specifically European perspective and excludes national rights instruments, which may miss how international rights are operationalized or challenged at the national or local level. Finally, the document-based approach does not include empirical perspectives (e.g., from athletes, policymakers, or marginalized groups), which could reveal how these rights function in practice and how their interpretation and application may be shaped by cultural context. Future research should examine domestic legal contexts, incorporate diverse stakeholder perspectives, and determine under what conditions participation restrictions are ethically justifiable beyond a rights-based framework. This paper has made significant progress by mapping the recognition of rights in relation to sport across international and European normative instruments, highlighting key inconsistencies and ambiguities, and reflecting on whether participation restrictions can be justified from a human rights perspective, thereby contributing to the literature and laying a foundation for future conceptual and normative work in this field.

## Data Availability

No datasets were generated or analysed during the current study.

## Footnotes

1 See Magavern et al. 2016, Magavern et al. 2017, Sutliff and Freeland 1995.

2 Examples of such restrictions can be found in Lim and Pranata 2020, Michelini et al. 2021, Angeli et al. 2021, Wolohan 1997, Sankaran et al. 1999, Maron 2015, Desomer et al. 2015, IFSC REDs Health Guidance for National Federations 2024. In a separate study (Sesa et al. forthcoming) we investigate how return-to-play following a concussion is currently being managed in football and how restrictions to participate in sport can be ethically justified.

3 Lim and Pranata 2020, Michelini et al. 2021, Angeli et al. 2021.

4 See Angeli et al. 2021, Pavlidis and Rowe 2021.

5 Wolohan 1997, Sankaran et al. 1999.

6 Maron 2015, see Desomer et al. 2015, Christou et al. 2021.

7 See IFSC REDs Health Guidance for National Federations 2024.

8 Neidecker et al. 2018.

9 Rugby AU Concussion Management Procedure 2025.

10 Holland 2023.

11 Ibid.

12 Dalglish et al. 2020.

13 Duval, Krüger, and Lindholm 2024.

14 Bowen 2009.

15 Veal 2022.

16 United Nations 1948.

17 United Nations 1966.

18 United Nations Committee on Economic, Social and Cultural Rights 2009.

19 Ibid., p.3.

20 Sivan and Veal 2021.

21 World Leisure Organization 2020.

22 Ireland-Piper and Weinert 2014.

23 Ibid.

24 Sivan and Veal 2021.

25 United Nations Committee on Economic, Social and Cultural Rights 2009, p.12.

26 Ibid.

27 Ibid.

28 World Leisure Organization 2020, p.2-3.

29 Ibid.

30 UN Committee on Economic, Social and Cultural Rights 2009, p.4-5.

31 UN Committee on Economic, Social and Cultural Rights 2009, p.18.

32 Ibid.

33 Kidd and Donnelly 2000.

34 Dionigi and Gard 2017.

35 Revised European Sports Charter 2001.

36 International Olympic Committee 1996.

37 International Olympic Committee 2025.

38 International Olympic Committee 2024.

39 Messing et al. 2021; Rodrigues, Bhattacharya and Cabete 2023.

40 See Rodrigues et al. 2023 and Veal 2022 for supporting arguments and see Lim 2012, Mitten and Davis 2008, and Krüger 2021 for opposing views.

41 Xanthaki 2024.

42 Ibid.

43 Ibid.

44 Holland 2023.

45 *See* European Sports Charter 2021.

46 Lord and Stein 2010, p.3.

47 Ibid.

48 Lim 2012.

49 Ibid.

50 Camporesi 2008.

51 Loland 2022.

52 See European Sports Charter 2021.

53 See Malm et al. 2019; Chen et al. 2019; Bachynski 2021.

54 Abanazir 2024.

55 Ibid, Jenny et al. 2016.

56 Xanthaki 2024.

57 See also Siracusa Principles 1984 (American Association for the International Commission of Jurists 1984).

58 Holland 2023.

59 Mitten and Davis 2008.

60 Yurlov 2015, p.124.

61 James and Osborn 2024, ch. 2.

62 Xanthaki 2024.
